# Bronchogenic Carcinoma with Cardiac Invasion Simulating Acute Myocardial Infarction

**DOI:** 10.1155/2016/7813509

**Published:** 2016-03-02

**Authors:** Anirban Das, Sibes K. Das, Sudipta Pandit, Rathindra Nath Karmakar

**Affiliations:** ^1^Department of Pulmonary Medicine, Murshidabad Medical College, Berhampore, West Bengal 742 101, India; ^2^Department of Pulmonary Medicine, Medical College, Kolkata, West Bengal 700 073, India; ^3^Department of Cardiology, Medical College, Kolkata, West Bengal 700 073, India

## Abstract

Cardiac metastases in bronchogenic carcinoma may occur due to retrograde lymphatic spread or by hematogenous dissemination of tumour cells, but direct invasion of heart by adjacent malignant lung mass is very uncommon. Pericardium is frequently involved in direct cardiac invasion by adjacent lung cancer. Pericardial effusion, pericarditis, and tamponade are common and life threatening presentation in such cases. But direct invasion of myocardium and endocardium is very uncommon. Left atrial endocardium is most commonly involved in such cases due to anatomical contiguity with pulmonary hilum through pulmonary veins, and in most cases left atrial involvement is asymptomatic. But myocardial compression and invasion by adjacent lung mass may result in myocardial ischemia and may present with retrosternal, oppressive chest pain which clinically may simulate with the acute myocardial infarction (AMI). As a result, it leads to misdiagnosis and delayed diagnosis of lung cancer. Here we report a case of non-small-cell carcinoma of right lung which was presented with asymptomatic invasion in left atrium and retrosternal chest pain simulating AMI due to myocardial compression by adjacent lung mass, in a seventy-four-year-old male smoker.

## 1. Introduction

Diversity in clinical presentations of bronchogenic carcinoma may result in diagnostic dilemma, delayed diagnosis, and sometimes misdiagnosis. Clinical manifestations of lung cancer are as follows: (a) features due to tumour itself, appearance of new symptoms or worsening of existing symptoms, (b) symptoms due to direct invasion or retrograde lymphatic spread of tumour cells to adjacent structures, (c) symptoms due to distant metastases caused by hematogenous spread or due to paraneoplastic syndrome caused by expression of different proteins, cytokines, hormones, and enzymes by tumour cells themselves, and finally (d) in very few cases, patients of lung cancer are asymptomatic, and lung malignancy is detected as incidental finding on chest radiograph, done for other indications. Pleura, pericardium, mediastinal, and hilar lymph nodes, great vessels, thoracic duct, esophagus, phrenic nerve, left recurrent laryngeal nerve, and sympathetic trunk are frequently involved by lung cancers due to direct invasion. But direct cardiac invasion by bronchogenic carcinoma is uncommon. Only 8–10% of all lung cancers present with invasion of heart, especially of left atrium [1], although 15–35% of patients of lung cancers have cardiac metastases, detected in autopsies [2]. Here, we report a rare case of non-small-cell carcinoma of lung, compressing adjacent ventricular wall, with invasion of left atrium, in a seventy-four-year-old male smoker.

## 2. Case Report

A 74-year-old normotensive, nondiabetic, male smoker (30-pack years) presented with progressively increasing shortness of breath, cough, white, mucoid expectoration, and retrosternal and right sided parasternal, oppressive chest pain for last 2 months. Chest pain and shortness of breath were increased in severity on exertion and were associated with palpitation, but there is no history of sweating, orthopnoea, paroxysmal nocturnal dyspnoea, unconsciousness, and convulsion. There was no radiation of chest pain. Chest pain was suddenly increased for last five days and the patient was admitted in the emergency department with a suspicion of acute myocardial infarction (AMI). There was no history of fever, hemoptysis, leg swelling, and facial puffiness. History of significant weight loss and anorexia were present. There was no history of household contact with the patient of sputum smear positive tuberculosis. The patient received formoterol + tiotropium metered dose inhaler (2 puffs once daily with spacer) and oral doxofylline (400 mg twice daily) for last five years, as he suffered from chronic bronchitis predominant chronic obstructive pulmonary disease (COPD) which resulted in persistent breathlessness, cough, and expectoration.

General survey revealed anemia and clubbing, but there was no cyanosis, edema, and engorged neck vein. There were multiple cervical and supraclavicular enlarged lymph nodes on left side, which were hard in consistency, discrete, nontender, and fixed to underlying structure, but not fixed to overlying skin, and there was no discharging sinus. His temperature was 37°C, respiratory rate 24 breaths/minute, pulse rate 120 beats/minute, regular blood pressure 94/64 mmHg, oxygen saturation in capillary blood (SpO_2_) 96% @ room air, and FiO_2_ 0.21.

**Figure 1:**
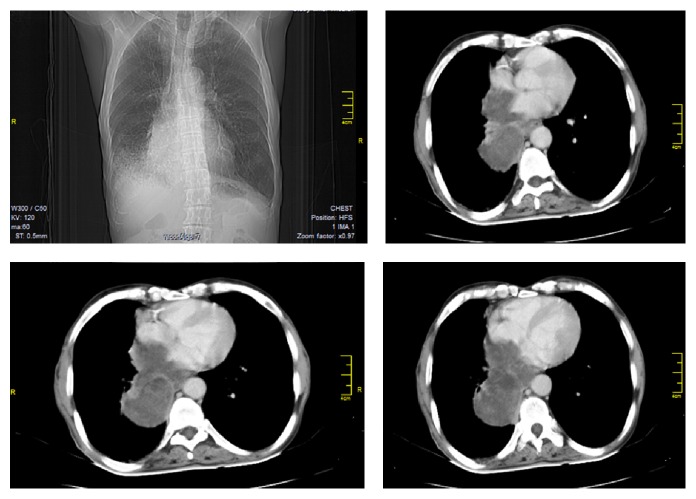
CECT thorax showing right lower lobe mass invading left atrium and compressing both ventricles.

**Figure 2:**
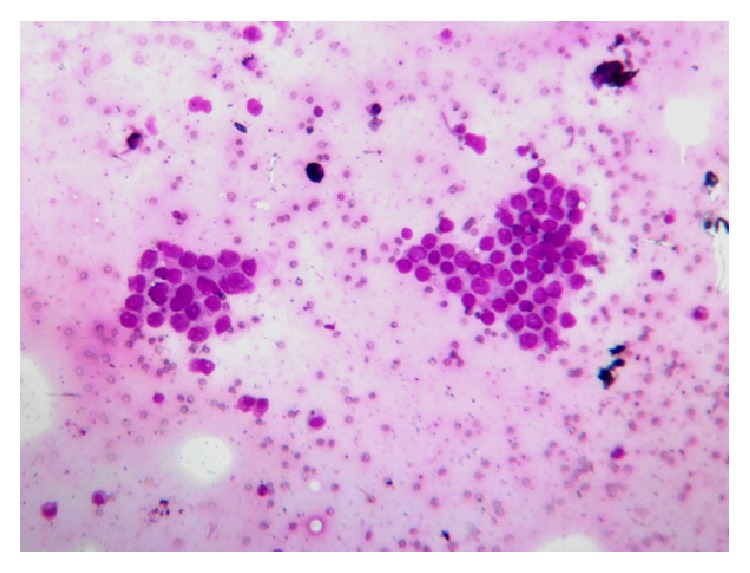
CT-guided FNAC of right lung mass showing non-small-cell carcinoma (MGG stain, 10x).

Examination of respiratory system revealed no abnormality, except vesicular breath sound with prolonged expiration on both sides, bilateral crackles, mainly over the bases of the lungs, and occasional wheezes. Examination of other system was normal. Complete hemogram showed anemia with hemoglobin concentration of 7.1 g/dL. Blood biochemistry was normal. Electrocardiogram (ECG) showed atypical ST segment elevation and T wave inversion in lateral chest leads (Lead V_4_, Lead V_5_, and Lead V_6_). Initial rapid troponin-*t*-test was inconclusive, but repeat one after 12 hours of first test was negative. Sputum for acid fast bacilli and Gram stain was negative, and pyogenic culture of sputum showed no growth. Chest X-ray posteroanterior (PA view) showed cardiothoracic ratio was more than 0.5; that is, size of the cardiac silhouette was increased, but lung parenchyma was normal. Contrast enhanced computed tomography (CECT) of thorax showed a large, nonhomogenous mass lesion in right lower lobe which invaded the left atrium and was compressing the adjacent ventricular walls (Figure 1). There was also mediastinal and hilar lymphadenopathy. CT-guided fine needle aspiration cytology (FNAC) showed discrete clusters of malignant epithelial cells which showed nuclear pleomorphism, hyperchromasia, high nucleocytoplasmic ratio, pale cytoplasm, and ill-defined cell boundary—suggestive of non-small-cell carcinoma, possibly squamous cell variety (Figure 2). FNAC of left sided supraclavicular lymph nodes showed metastatic squamous cell carcinoma. Sputum for malignant cell was negative and fibre optic bronchoscopy did not show any abnormality. Echocardiogram showed a large echogenic mass of 15 × 12 mm, attached to the roof of the left atrium adjacent to right pulmonary venous openings, encroaching to interatrial septum, an extension of the tumour from adjacent lung parenchyma. Ejection fraction was 66%. Ultrasound of abdomen and CECT scan of brain were normal. Hence the diagnosis was a non-small-cell carcinoma of lower lobe of right lung with invasion of the left atrium and compression of adjacent ventricular wall (T4 disease) with contralateral supraclavicular metastatic lymphadenopathy (N3 disease). First cycle chemotherapy regimen comprising intravenous cisplatin (100 mg on day 1) and etoposide (100 mg on days 1, 2, and 3) was given, but unfortunately he died just after completion of first cycle.

## 3. Discussion

Incidence of metastatic tumours of the heart is more than that of primary cardiac neoplasms. Cardiac myxoma is the most common primary tumour of heart. Metastases to heart occur in 1.5–21% of all malignant tumours [3]. Lung cancers are most common primary malignant tumours which metastasize to heart. Other malignancies causing cardiac metastasis are melanoma, lymphoma, breast cancer, leukemia, and stomach cancer [4, 5].

Spread of lung cancers to heart may occur due to retrograde lymphatic spread, hematogenous dissemination, direct invasion, or transvenous extension. Pericardial involvement most commonly occurs due to lymphatic spread, whereas hematogenous spread preferentially causes myocardial tumour deposition. Endocardial involvement is very rare. Although heart is very close structure to lung parenchyma, lung cancers spread to heart most commonly by lymphatic route, rather than direct invasion. Left atrium is anatomically contiguous structure to the lung hilum via pulmonary veins, and it explains why left atrium is most commonly involved compared with right atrium and both ventricles by direct invasion of central lung tumours [6]. In our case, right lower lobe lung mass was compressing the posterior walls of both ventricles and invading the roof of the left atrium, probably by direct invasion through the superficial pulmonary veins.

In most cases, cardiac metastases are asymptomatic, detected on autopsies after death. But in few cases it may be first manifestation, even sole presentation of lung cancers. Pericardial invasion results in hemorrhagic or straw-coloured pericardial effusion or constrictive pericarditis. Neoplastic pericardial tamponade in most cases is the ultimate cause of death. Myocardial invasion results in myocardial ischemia and angina pectoris, brady- or tachyarrhythmias (e.g., unexplained tachycardia, atrial flutter, atrial fibrillation, heart block, complete or incomplete, atrioventricular rhythm, and premature beats), and congestive cardiac failure [7]. Conduction abnormalities of the heart occur as lung tumours infiltrating the cardiac conduction tissues located within the interatrial septum and interventricular septum. In our case, retrosternal oppressive chest pain was probably due to myocardial ischemia caused by compression of posterior wall of heart by the lung mass. Lung mass itself may be an additional factor for development of dull aching chest pain. Intracavitary cardiac metastases from lung cancers in most cases are asymptomatic. But in some cases it may produce soft, systolic, or diastolic murmurs. Diastolic murmurs are caused by tumour-related obstruction of the left or right ventricular filling, and systolic murmurs are due to interference with the closure of atrioventricular valves or the narrowing of the ventricular outflow tract. In our case, left atrial invasion by tumour did not produce any symptom. Hence, clinically our initial provisional diagnosis was acute myocardial infarction with the following differentials: angina pectoris, pulmonary thromboembolism, pneumothorax, musculoskeletal pain involving chest wall, gastroesophageal reflux with esophageal spasm, lung cancer, and pneumonia with pleural involvement, and so forth.

CECT thorax or magnetic resonance imaging (MRI) of mediastinum distinctly delineates the morphological appearance and the degree of infiltration into heart by juxtacardiac lung tumour [8]. Two-dimensional echocardiogram also detects intracavitary extension of lung tumour as well as pericardial and myocardial involvement. Change in the electrocardiogram is nonspecific; in most cases it is normal. However, the following abnormalities may be seen: persistent ST elevation, persistent T wave inversion, conduction defects, low-voltage QRS, and so forth [4].

In most of the cases, cardiac metastases are seen in advanced lung cancer with or without distant metastases. In cases of distant metastases, heart is central site from which generalized tumour dissemination occurs. Treatment of cardiac invasion by lung cancer is palliative; chemo- or radiotherapy is main treatment option. In our case, first cycle chemotherapy was given, but the patient died just afterwards. There was no option for surgical resection, because the disease was in advanced stage (stage IIIB), as evidenced by metastasis to contralateral supraclavicular lymph nodes.
